# Supplementary material for: Altered TMPRSS2 usage by SARS-CoV-2 Omicron impacts infectivity and fusogenicity
Source: Nature. 2022 Feb 1;603(7902):706–14. doi: 10.1038/s41586-022-04474-x (PMC8942856; doi:10.1038/s41586-022-04474-x)
Supplement: Supplementary file 1 — Uncropped western blots for Fig. 2; the FACS gating strategy for Extended Data Fig.8; and the uncropped western blots for Extended Data Fig. 6. [file 41586_2022_4474_MOESM1_ESM.pdf]

nature portfolio

https://doi.org/10.1038/s41586-022-04474-x

Supplementary information

Altered TMPRSS2 usage by SARS-CoV-2 Omicron impacts infectivity and fusogenicity

In the format provided by the authors and unedited

Supplementary information: Meng et al, Nature 2022

- Page 1: uncropped western blots figure 2
- Page 2: FACS gating strategy Extended Data Figure 2
- Page 3: uncropped western blots Extended Data Figure 6

SI for Figure 2f&g

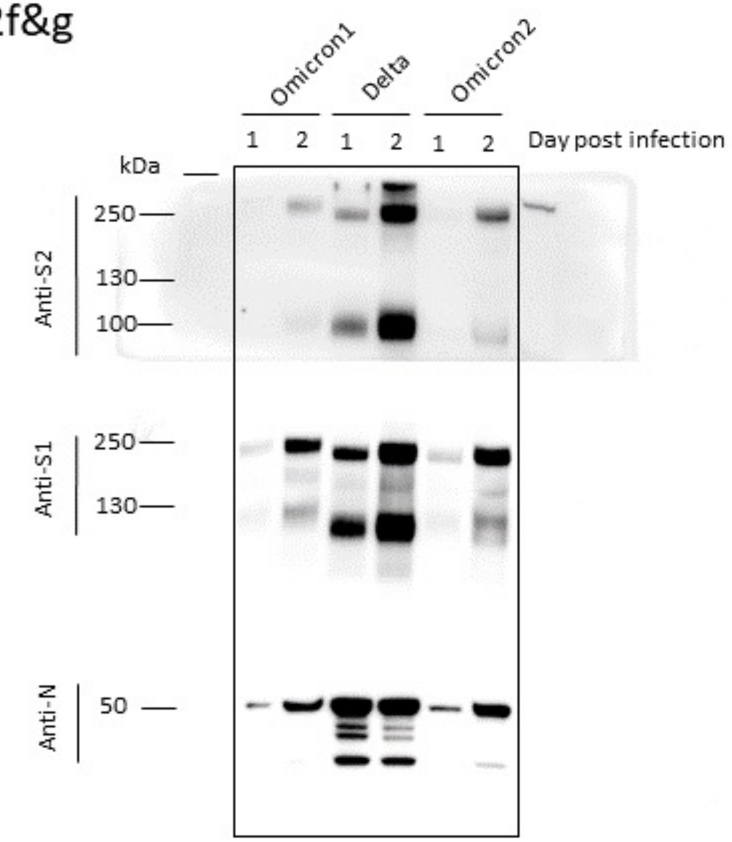

Cut the blot into two halves around 75 KDa
Probed S2 first then stripped for S1
Probed N

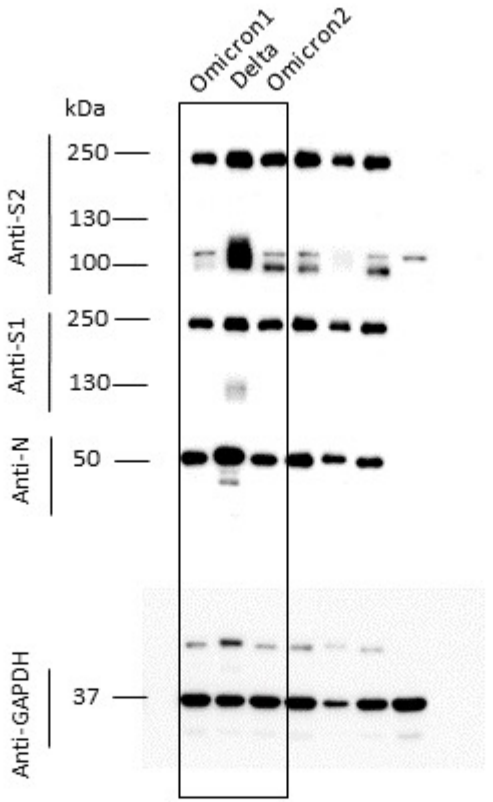

Cut the blot into two halves around 75 KDa
Probed S2 first then stripped for S1
Probed N first then GAPDH

SI for ED Fig 2: gating strategy for ACE2 binding and Spike expression analyses

ACE2 binding

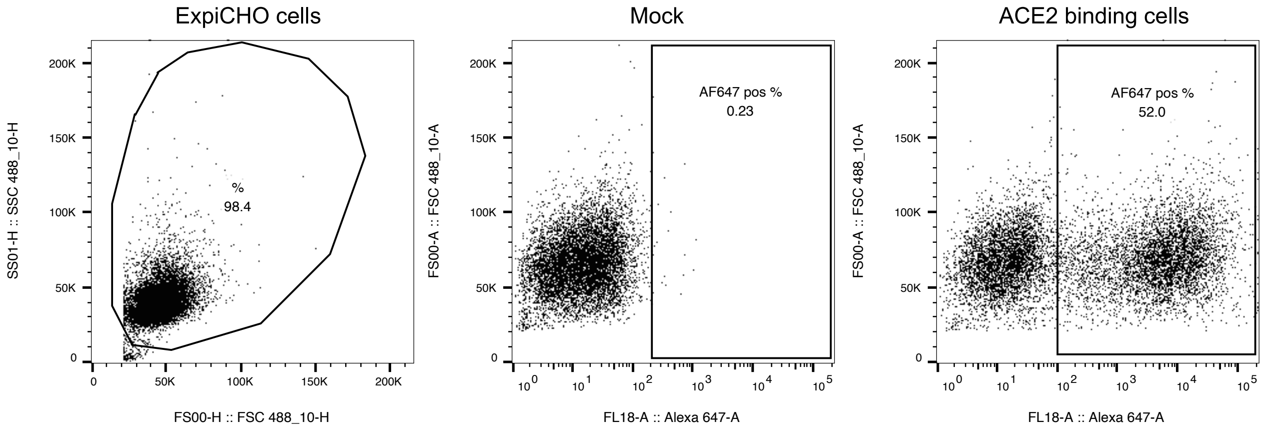

Spike expression

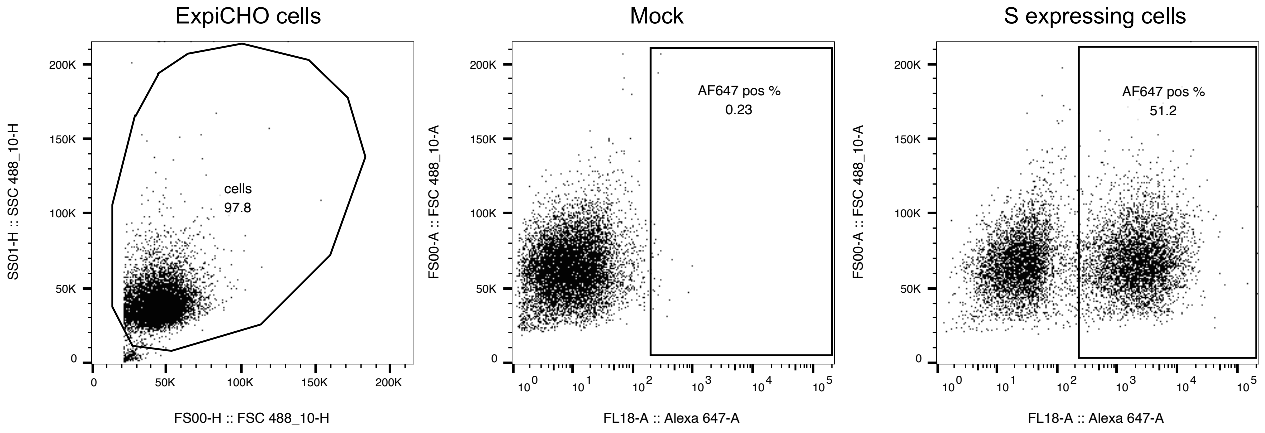

SI for ED Fig 6

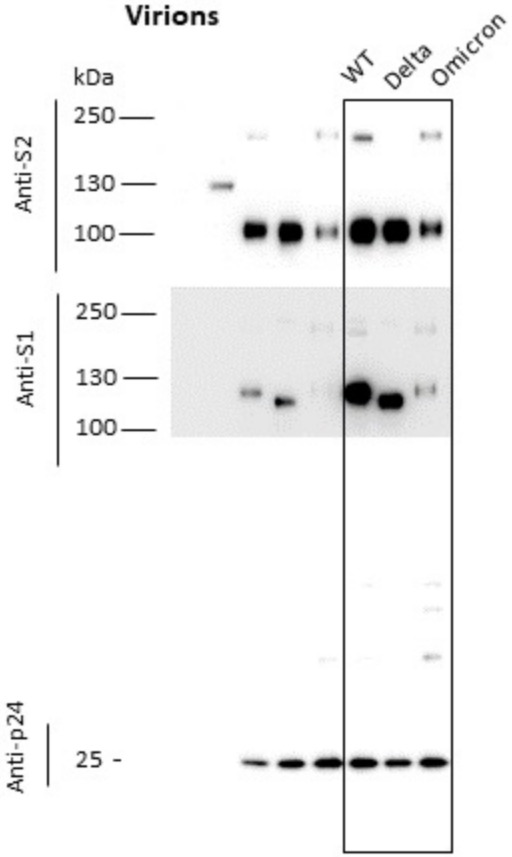

Cut blot into two halves around 75 kDa;
First blotted with anti-S2 antibody and then stripped and reblotted with anti-S1 antibody;
The other half blotted with anti-p24 antibody

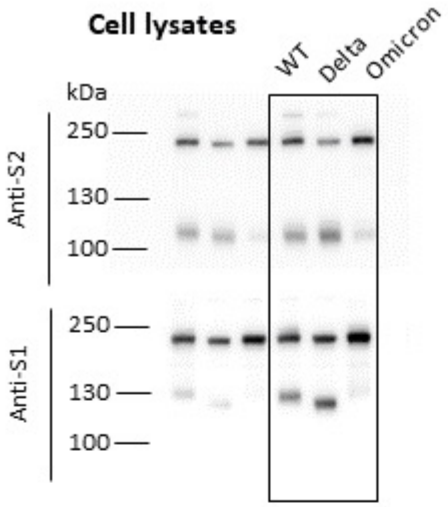

First blotted with anti-S2 antibody and then stripped and reblotted with anti-S1 antibody.
