# Supplementary material for: Altered TMPRSS2 usage by SARS-CoV-2 Omicron impacts infectivity and fusogenicity
Source: Nature. 2022 Feb 1;603(7902):706–14. doi: 10.1038/s41586-022-04474-x (PMC8942856; doi:10.1038/s41586-022-04474-x)
Supplement: Supplementary file 2 — Reporting Summary [file 41586_2022_4474_MOESM2_ESM.pdf]

nature portfolio

Corresponding author(s): Sato, Gupta

Last updated by author(s): Dec 24, 2021

Reporting Summary

Nature Portfolio wishes to improve the reproducibility of the work that we publish. This form provides structure for consistency and transparency in reporting. For further information on Nature Portfolio policies, see our Editorial Policies and the Editorial Policy Checklist.

Statistics

For all statistical analyses, confirm that the following items are present in the figure legend, table legend, main text, or Methods section.

n/a	Confirmed	
☐	☒	The exact sample size (n) for each experimental group/condition, given as a discrete number and unit of measurement
☐	☒	A statement on whether measurements were taken from distinct samples or whether the same sample was measured repeatedly
☐	☒	The statistical test(s) used AND whether they are one- or two-sided<br>*Only common tests should be described solely by name; describe more complex techniques in the Methods section.*
☒	☐	A description of all covariates tested
☒	☐	A description of any assumptions or corrections, such as tests of normality and adjustment for multiple comparisons
☐	☒	A full description of the statistical parameters including central tendency (e.g. means) or other basic estimates (e.g. regression coefficient) AND variation (e.g. standard deviation) or associated estimates of uncertainty (e.g. confidence intervals)
☐	☒	For null hypothesis testing, the test statistic (e.g. F, t, r) with confidence intervals, effect sizes, degrees of freedom and P value noted<br>*Give P values as exact values whenever suitable.*
☒	☐	For Bayesian analysis, information on the choice of priors and Markov chain Monte Carlo settings
☒	☐	For hierarchical and complex designs, identification of the appropriate level for tests and full reporting of outcomes
☐	☒	Estimates of effect sizes (e.g. Cohen's d, Pearson's r), indicating how they were calculated

Our web collection on statistics for biologists contains articles on many of the points above.

Software and code

Policy information about availability of computer code

Data collection	*Provide a description of all commercial, open source and custom code used to collect the data in this study, specifying the version used OR state that no software was used.*
Data analysis	Prism 9, Excel, GROMACS2018; FloJo, Fiji (v1.49) software, ImageJ, geneious prime, scanpy, Alphafold, GISAID and EpiCoV websites

For manuscripts utilizing custom algorithms or software that are central to the research but not yet described in published literature, software must be made available to editors and reviewers. We strongly encourage code deposition in a community repository (e.g. GitHub). See the Nature Portfolio guidelines for submitting code & software for further information.

Data

Policy information about availability of data

All manuscripts must include a data availability statement. This statement should provide the following information, where applicable:

- Accession codes, unique identifiers, or web links for publicly available datasets
- A description of any restrictions on data availability
- For clinical datasets or third party data, please ensure that the statement adheres to our policy

The raw data in this study are available from the corresponding authors with no restrictions upon reasonable request.

Field-specific reporting

Please select the one below that is the best fit for your research. If you are not sure, read the appropriate sections before making your selection.

☒ Life sciences ☐ Behavioural & social sciences ☐ Ecological, evolutionary & environmental sciences

For a reference copy of the document with all sections, see nature.com/documents/nr-reporting-summary-flat.pdf

Life sciences study design

All studies must disclose on these points even when the disclosure is negative.

Sample size	Our sample sizes were based on our previous in vitro SARS-CoV-2 work, and based on practical availability of sera from participants.
Data exclusions	Individuals positive for N antibody were excluded from analysis in neutralisation experiments and data with and without the exclusions are presented in the figures
Replication	experiments were done at least twice as biological replicates
Randomization	we did not use randomisation in our lab experimental designs
Blinding	we did not use blinding in our lab experimental designs

Reporting for specific materials, systems and methods

We require information from authors about some types of materials, experimental systems and methods used in many studies. Here, indicate whether each material, system or method listed is relevant to your study. If you are not sure if a list item applies to your research, read the appropriate section before selecting a response.

Materials & experimental systems

n/a	Involved in the study	
☐	☒	Antibodies
☐	☒	Eukaryotic cell lines
☒	☐	Palaeontology and archaeology
☒	☐	Animals and other organisms
☐	☒	Human research participants
☒	☐	Clinical data
☒	☐	Dual use research of concern

Methods

n/a	Involved in the study	
☒	☐	ChIP-seq
☐	☒	Flow cytometry
☒	☐	MRI-based neuroimaging

Antibodies

Antibodies used

For Western blot:
Rabbit anti-SARS-CoV-2 S polyclonal antibody (PA1-41165, Thermofisher, 1:2,000),
Rabbit anti-SARS-CoV-2 N monoclonal antibody (clone HL344, GeneTex, Cat# GTX635679, 1:2,000),
Mouse anti-SARS-CoV-2 S1 monoclonal antibody (MAB105403, R&D systems, 1:2,000),
Rabbit anti-GAPDH polyclonal antibody (10494-1-AP, Proteintech, 1:5,000),
Mouse anti-HIV p24 monoclonal antibody (NIBSC, ARP313, 1:20,000)
Horseradish peroxidase (HRP)-conjugated Goat anti-rabbit IgG antibody (Cell Signaling, 7074, 1:5,000),
HRP-conjugated anti-mouse IgG antibody (Cell Signaling, 7076, 1:5,000).

For neutralisation assay:
Casirivimab and Imdevimab

For IF
Mouse anti-SARS-CoV-2 Spike monoclonal antibody (clone1A9, GeneTex, GTX632604,)
anti-GM130[EP892Y]-cis-Golgi (ab52649, 1:250)
phalloidin 647 (Thermofisher, A22287, 1:1000)
Goat anti-Rabbit IgG (H+L) Cross-Adsorbed Secondary Antibody, Alexa Fluor 594 (Thermofisher, A-11012, 1:1,000)
Goat anti-Mouse IgG (H+L) Cross-Adsorbed Secondary Antibody, Alexa Fluor 488 (Thermofisher, A-11001, 1:1,000)

For FACS
Alexa Fluor647 Goat Anti-Human IgG secondary Ab (1.5 mg/ml) (Jackson Immunoresearch)
rabbit anti-SARS-CoV-2 S S1/S2 polyclonal antibody (Thermo Fisher Scientific, Cat# PA5-112048, 1:100).
Normal rabbit IgG (SouthernBiotech, Cat# 0111-01, 1:100)
APC-conjugated goat anti-rabbit IgG polyclonal antibody (Jackson ImmunoResearch, Cat# 111-136-144, 1:50)

Validation

Rabbit anti-SARS-CoV-2 S polyclonal antibody (PA1-41165, Thermofisher, 1:2,000),
Rabbit anti-SARS-CoV-2 N monoclonal antibody (clone HL344, GeneTex, Cat# GTX635679, 1:2,000),
Mouse anti-SARS-CoV-2 S1 monoclonal antibody (MAB105403, R&D systems, 1:2,000),
These products were validated by putting negative controls of the antigens in western blotting. We verified that the bands detected at appropriated sizes were not observed in the samples from the antigen-negative cells.

Rabbit anti-GAPDH polyclonal antibody (10494-1-AP, Proteintech, 1:5,000). This antibody has been tested as stated on the supplier's website in human placenta tissue, Raji cells, HepG2 cells, K-562 cells, mouse heart tissue, A549 cells, PC-13 cells, arabidopsis whole plant tissue, corn whole plant tissue, mouse brain tissue, HEK-293 cells, HeLa cells, rat brain tissue, mouse skin tissue, RAW 264.7 cells, C6 cells, Jurkat cells, NIH/3T3 cells

Horseradish peroxidase (HRP)-conjugated Goat anti-rabbit IgG antibody (Cell Signaling, 7074, 1:5,000),
HRP-conjugated anti-mouse IgG antibody (Cell Signaling, 7076, 1:5,000).
Alexa Fluor647 Goat Anti-Human IgG secondary Ab (1.5 mg/ml) (Jackson Immunoresearch)
anti-GM130[EP892Y]-cis-Golgi (ab52649, 1:250)
phalloidin 647 (Thermofisher, A22287, 1:1000)
Goat anti-Rabbit IgG (H+L) Cross-Adsorbed Secondary Antibody, Alexa Fluor 594 (Thermofisher, A-11012, 1:1,000)
Goat anti-Mouse IgG (H+L) Cross-Adsorbed Secondary Antibody, Alexa Fluor 488 (Thermofisher, A-11001, 1:1,000)
These products were thoroughly validated by the providers and work optimally with the protocol for Western blotting or FACS or IF, as described in the Method section. We ensured the results are accurate and reproducible.

Casirivimab and Imdevimab were generated as described in the Method section. The nucleotide sequences of their templates were verified by Sanger sequencing.

Eukaryotic cell lines

Policy information about cell lines

Cell line source(s)

HEK293T cells (a human embryonic kidney cell line; ATCC CRL-3216)
HOS-ACE2/TMPRSS2 cells (HOS cells stably expressing human ACE2 and TMPRESS2) (Saito et al., Nature, 2021)
VeroE6/TMPRSS2 cells [an African green monkey (Chlorocebus sabaeus) kidney cell line; JCRB1819]
Calu-3 cells (a human lung epithelial cell line; ATCC HTB-55 or a gift from Paul Lehner)
H1299 a gift from Simon Cook
HeLa-ACE2 a gift from James Voss
CaCo2 a gift from Ian Goodfellow
A549-ACE2/TMPRSS2 from Massimo Palmarini
293T (CRL-3216)
293T-ACE2/TMPRSS2, 293T-ACE2ΔTMPRSS2, 293T-TMPRSS2, 293T-GFP11 and Vero -GFP1-10 were provided by Leo James
Vero-ACE2/TMPRSS2 from Emma Thomson
ExpiCHO cells from Davide Corti
A549-ACE2-TMPRSS2-based luminescent reporter cells from Nick Matheson

Authentication

HEK293T cells, H1299 and Calu-3 cells were authenticated by ATCC.
VeroE6/TMPRSS2 cells was authenticated by JCRB Cell Bank.
HOS-ACE2/TMPRSS2 cells Saito et al., Nature, 2021) was not authenticated.

Mycoplasma contamination

All cell lines were regularly tested for mycoplasma contamination by using PCR and were confirmed to be mycoplasma-free.

Commonly misidentified lines
(See ICLAC register)

No commonly misidentified cell lines were used.

Human research participants

Policy information about studies involving human research participants

Population characteristics

Vaccine sera were collected from fourteen vaccinees four weeks after the second vaccination with BNT162b2 (Pfizer-BioNTech) (average age: 46, range: 38-55, 21% male),

ten vaccinees four weeks after the second vaccination with mRNA-1273 (Moderna) (average age: 26, range: 21-37, 40% male),

nine vaccinees 4-6 weeks after the second vaccination with CoronaVac (Sinovac) (44% male).

Delta convalescent Delta sera were collected from ten vaccine-naive individuals who had infected with Delta variant (B.1.617.2 or AY.29) (average age: 46, range: 22-63, 70% male).

Recruitment

The voluntary donors were recruited at Kyoto University (BNT162b2 and mRNA-1273 vaccinees),

Universidad San Francisco de Quito (CoronaVac vaccinees),

	Kuramochi Clinic Interpark (Delta convalescents)<br><br>regardless of age, sex, gender, race, ethnicity, or other characteristics. Written informed consent was obtained from the voluntary donor.<br><br>Community vaccinated participants were also recruited by the NIHR BioResource
Ethics oversight	All protocols involving specimens from human subjects recruited at<br><br>Kyoto University,<br><br>Kuramochi Clinic Interpark<br><br>Universidad San Francisco de Quito<br><br>were reviewed and approved by the Institutional Review Boards of<br><br>Kyoto University (approval ID: G0697),<br><br>Kuramochi Clinic Interpark (approval ID: G2021-004)<br><br>Universidad San Francisco de Quito (approval ID: CEISH P2020-022IN), and the Ecuadorian Ministry of Health (approval IDs: MSP-CGDES-2020-0121-O and MSP-CGDES-061-2020).<br><br>Ethical approval for study of vaccine elicited antibodies in sera from vaccinees was obtained from the East of England – Cambridge Central Research Ethics Committee Cambridge (REC ref: 17/EE/0025).<br><br>Ethical approval for use of human tissue for measurement of ACE2 and TMPRSS2 levels was obtained as follows: the samples of human upper and lower airways were obtained from the lungs of a multiorgan donor whose lungs were deemed unsuitable for clinical transplantation and after their next of kin consented to their use in research. The studies using human donor lungs tissue were approved by National Research Ethics Committee (NREC) 16/NE/0230.

Note that full information on the approval of the study protocol must also be provided in the manuscript.

Flow Cytometry

Plots

Confirm that:

☒ The axis labels state the marker and fluorochrome used (e.g. CD4-FITC).

☒ The axis scales are clearly visible. Include numbers along axes only for bottom left plot of group (a 'group' is an analysis of identical markers).

☒ All plots are contour plots with outliers or pseudocolor plots.

☒ A numerical value for number of cells or percentage (with statistics) is provided.

Methodology

Sample preparation	HEK293 cells were cotransfected with 400 ng of D614G S or D614G/P681R expression plasmids and 400 ng pDSP1-7 using TransIT-LT1 (Takara, Cat# MIR2300).
Instrument	FACS Canto II instrument (BD Biosciences)
Software	FlowJo
Cell population abundance	n/a (cell sorting was not conducted)
Gating strategy	Extended Data Fig. 8.<br>The cells were gated in the FSC/SSC plot, then the mean fluorescence intensity of surface S protein (APC) was measured. The boundary between S-positive and S-negative was defined by using the cells that the S-expression plasmid was not transfected (i.e., S-negative cells).

☒ Tick this box to confirm that a figure exemplifying the gating strategy is provided in the Supplementary Information.
